# Supplementary material for: Antidiabetic Effect of Casein Glycomacropeptide Hydrolysates on High-Fat Diet and STZ-Induced Diabetic Mice via Regulating Insulin Signaling in Skeletal Muscle and Modulating Gut Microbiota
Source: Nutrients. 2020 Jan 15;12(1):220. doi: 10.3390/nu12010220 (PMC7019650; doi:10.3390/nu12010220)
Supplement: Supplementary file 1 [file nutrients-12-00220-s001.pdf]

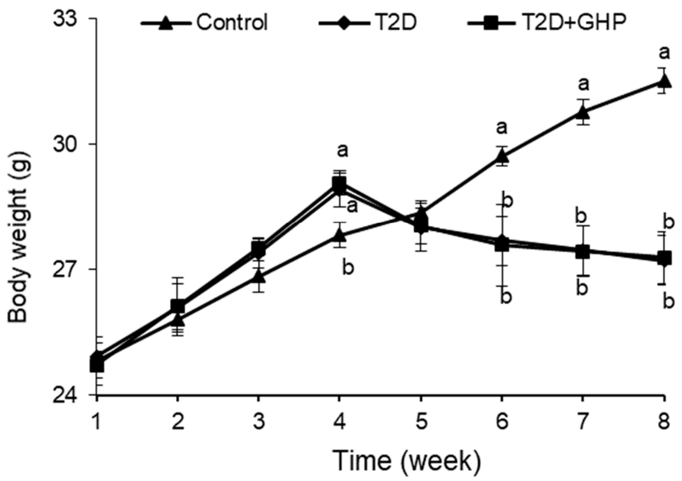

Figure S1 Body weight before GHP administration. Mice in T2D and T2D+GHP groups were fed high-fat diet for eight weeks and were injected with STZ in the fifth week to induce diabetes. Values with different letters (a-b) indicate significantly difference ($p<0.05$).
